# CARs derived from broadly neutralizing, human monoclonal antibodies identified by single B cell sorting target hepatitis B virus-positive cells

**DOI:** 10.3389/fimmu.2024.1340619

**Published:** 2024-04-22

**Authors:** Sophia Schreiber, Lisa S. Dressler, Eva Loffredo-Verde, Theresa Asen, Stephanie Färber, Wenshi Wang, Tanja Groll, Anindita Chakraborty, Fenna Kolbe, Christoph Kreer, Anna D. Kosinska, Sylvain Simon, Stephan Urban, Florian Klein, Stanley R. Riddell, Ulrike Protzer

**Affiliations:** ^1^ Institute of Virology, School of Medicine, Technical University of Munich / Helmholtz Munich, Munich, Germany; ^2^ German Center for Infection Research, Munich Partner Site, Munich, Germany; ^3^ Department of Infectious Diseases, Molecular Virology, University Hospital, Heidelberg, Germany; ^4^ Institute of Pathology, School of Medicine, Technical University of Munich, Munich, Germany; ^5^ Laboratory of Experimental Immunology, Institute of Virology, University of Cologne, Faculty of Medicine and University Hospital of Cologne, Cologne, Germany; ^6^ Translational Science and Therapeutics Division, Fred Hutchinson Cancer Center, Seattle, WA, United States

**Keywords:** human monoclonal antibody, broad neutralization, single B cell sorting, hepatitis B virus envelope proteins, HBsAg, chimeric antigen receptor, tonic signaling, linear epitope

## Abstract

To design new CARs targeting hepatitis B virus (HBV), we isolated human monoclonal antibodies recognizing the HBV envelope proteins from single B cells of a patient with a resolved infection. HBV-specific memory B cells were isolated by incubating peripheral blood mononuclear cells with biotinylated hepatitis B surface antigen (HBsAg), followed by single-cell flow cytometry-based sorting of live, CD19^+^ IgG^+^ HBsAg^+^ cells. Amplification and sequencing of immunoglobulin genes from single memory B cells identified variable heavy and light chain sequences. Corresponding immunoglobulin chains were cloned into IgG1 expression vectors and expressed in mammalian cells. Two antibodies named 4D06 and 4D08 were found to be highly specific for HBsAg, recognized a conformational and a linear epitope, respectively, and showed broad reactivity and neutralization capacity against all major HBV genotypes. 4D06 and 4D08 variable chain fragments were cloned into a 2^nd^ generation CAR format with CD28 and CD3zeta intracellular signaling domains. The new CAR constructs displayed a high functional avidity when expressed on primary human T cells. CAR-grafted T cells proved to be polyfunctional regarding cytokine secretion and killed HBV-positive target cells. Interestingly, background activation of the 4D08-CAR recognizing a linear instead of a conformational epitope was consistently low. In a preclinical model of chronic HBV infection, murine T cells grafted with the 4D06 and the 4D08 CAR showed on target activity indicated by a transient increase in serum transaminases, and a lower number of HBV-positive hepatocytes in the mice treated. This study demonstrates an efficient and fast approach to identifying pathogen-specific monoclonal human antibodies from small donor cell numbers for the subsequent generation of new CARs.

## 1 Introduction

Chronic HBV infection remains a global health problem. Despite the availability of an effective vaccine, approximately 296 million people are chronically infected worldwide and carry an increased risk of developing liver cirrhosis and hepatocellular carcinoma (1). In contrast to other infectious diseases such as malaria, tuberculosis, or HIV, viral hepatitis-related mortality rates are still on the rise (2), amounting to 820,000 HBV-associated deaths per year (1). Current standard therapy using nucleos(t)ide analogs suppresses new virion production and HBV DNA and reduces liver inflammation but has little or no effect on the expression of the small HBV envelope (HBVenv) protein (S) (3). Thus, viral eradication is rarely achieved, and a significant cancer risk remains (4, 5). This is why there is an ongoing need to research novel treatment options for the cure of HBV infection.

A key characteristic of chronic HBV infection is a scarce and dysfunctional T-cell response (6). In contrast, an acute and resolving course of infection is marked by a strong and polyfunctional virus-specific T-cell response (7). Furthermore, clinical reports showed that transferring pre-existing HBV immunity through bone marrow transplantation for lymphoma therapy cleared the infection in the receiving patients (8). Therefore, reconstituting HBV-specific T-cell immunity via adoptive transfer of genetically redirected T cells emerged as a promising therapeutic strategy in recent years (9). To date, both HBV-specific T-cell receptors (TCRs) and chimeric antigen receptors (CARs) have shown encouraging results *in vitro* and *in vivo* (9–13). While TCRs are therapeutically confined to a limited number of patients given their HLA restriction, CARs harbor the advantage of targeting surface-bound antigens on target cells in an MHC-independent fashion (9). In our laboratory, we previously generated an HBV-specific S-CAR based on the single-chain variable fragment (scFv) C8 that was identified from a phage display library generated out of peripheral blood mononuclear cells (PBMC) of HBV-vaccinated donors (14). This S-CAR was shown to recognize all HBVenv proteins on the surface of infected cells (15), as the S-domain is also contained in the middle (M) and large (L) HBVenv proteins. Furthermore, it proved functional *in vitro* and in mouse models of chronic HBV infection (10, 11). Still, it showed limitations in terms of sensitivity, in particular, compared to HBV-specific TCRs, and it induces a certain level of tonic signaling, a problem known for several CARs targeting a variety of antigens.

Therefore, in the present study, we set out to generate an alternative set of CAR constructs for adoptive T-cell therapy of HBV infection. We started by searching for novel, recombinant, and fully human monoclonal antibodies (mAbs) against HBV by single B-cell sorting technology (16–18). For this purpose, we worked with PBMC from a donor with resolved HBV infection, as the chances of isolating high-quality antibodies were higher than with samples from vaccinated individuals. We successfully cloned two antibodies named 4D06 and 4D08, which displayed high affinities in a nanomolar range, broad neutralizing capacity, and the potential to recognize all known HBV genotypes. These antibodies were then cloned as scFv into a 2^nd^ generation CAR format with intracellular CD28 and CD3zeta signaling domains and compared to the existing C8-CAR in terms of expression and functionality on HBsAg and HBV-expressing target cells.

## 2 Materials and methods

### 2.1 Identification of HBsAg-specific B cell clones and antibodies

PBMC were isolated via a standard Ficoll gradient. Informed consent in writing was obtained from each donor. B cells were enriched from PBMC using Dynabeads Untouched Human B cells Kit (Thermo Fisher). For further analysis, B cells were incubated with human Fc block (BD Biosciences) for 10’ at RT, followed by biotinylated HBsAg for 30’ on ice (HBsAg by Roche Diagnostics; EZ-Link Sulfo-NHS-Biotinylation kit, Thermo Fisher). Memory B cell staining: 30’ on ice in PBS+0.5% BSA with live-dead stain (Thermo Fisher), anti-CD19-eF450 (eBioscience), anti-IgG-Pe-Cy7 (BD) and Streptavidin-APC (eBioscience). Cells were analyzed via flow cytometry on Cytoflex S (Beckman Coulter) or sorted with MoFlo II (Beckman Coulter) into lysis buffer, i.e. PBS+12U RNasin (Promega), 100 mM DTT (Thermo Fisher). PCR plates were sealed and stored at -80°C. IgG gene identification via PCR and sequencing from single B cells was performed as described elsewhere (16).

### 2.2 B cell ELISpot

PBMC were stimulated with 1 µg/mL R848 and 10 ng/mL IL2 (MabTech) at 2x10^6^ cells/mL for 5d at 37°C. ELISpot plates (Merck) were activated and coated ON at 4°C with 2 µg/mL HBsAg (Roche Diagnostics), washed 1x with PBS, and blocked with RPMI for 2h at RT. Stimulated PBMC were added to ELISpot plates at 1-2x10^5^/well in RPMI and incubated for 18h at 37°C. B cells were subsequently removed, and plates were washed 5x with PBS. 1 µg/ml detection antibody MT78/145 (MabTech) was added in PBS+0.5%BSA for 2h at RT. After washing, Streptavidin-HRP (MabTech) in PBS+0.5%BSA was added for 1h at RT, followed by TMB (MabTech). Spot development was stopped by rinsing with H_2_O, and spot numbers were quantified with ImmunoSpot Reader (CTL Europe).

### 2.3 Antibody expression and ELISA

HEK293 cells were transiently transfected with heavy and light chains using Fugene HD transfection reagent (Promega). 3 days post-transfection, the supernatant was analyzed via sandwich ELISA. The following reagents were used for detecting hIgG: goat anti-human IgG for coating 1:500 in PBS (Thermo Fisher), PBS+5% BSA for blocking, and PBS+0.05% Tween-20 for washing as well as polyclonal goat anti-human IgG-HRP 1:1000 in PBS (Sigma Aldrich) and TMB (Thermo Fisher) for detection. For the detection of HBsAg-specific antibodies, pre-coated Protein G ELISA plates (Pierce, Thermo Fisher) were used. After adding samples, HBsAg-biotin was added 1:200 in PBS, and 1:500 Avidin-HRP in PBS (eBioscience) and TMB (MabTech) were used for development. Absorbance at 450 nm was measured with Tecan Reader (Tecan Group AG).

### 2.4 Epitope determination with dot blot and Western blot

An Immuno-Blot PVDF membrane (Biorad) was activated and calibrated with pure methanol and PBS for the dot blot. Drops of 2 µL were placed on the membranes (3 drops per piece) containing HBsAg (1 µg/mL) either diluted in PBS (non-reducing condition) or heated for 10’ at 95°C in dot blot buffer (0.125 M Tris-HCl, 4% SDS, 20% Glycerol, 10% β-mercaptoethanol) (reducing condition). After complete drying, the membranes were blocked with 5% milk in TBS-T buffer for 1h at RT. Membranes were then transferred into 5% milk TBS-T solutions containing the primary antibodies 4D06, 4D08, or HB-1 with a concentration of 1 µg/ml. After incubation for 1.5h at RT, the membranes were washed 3x for 10’ in TBS-T buffer and placed into 5% milk in TBS-T buffer containing secondary antibody goat anti-human IgG HRP (Sigma Aldrich) diluted 1:10,000 for 1h at RT. After final washing, an ECL detection solution (GE Healthcare) was added to develop and analyze the blots.

The HBsAg Western blot was performed similarly. HBsAg was heated for 20’ at 95°C in dot blot buffer and loaded onto a 12% SDS-PAGE gel. Separated proteins were wet-blotted onto an Immuno-Blot PVDF membrane (Biorad) and blocked in the same way as described above for the dot blot. The antibodies were used at 1 µg/mL diluted in 5% milk in TBS-T buffer. All further steps were performed as in the dot blot.

### 2.5 HBsAg ELISA

ELISA plates were coated ON at 4°C with 1 µg/mL HBsAg. Plates were washed 4x times with PBS+0.05% Tween20 (Sigma Aldrich) and blocked with 5% BSA in PBS for 1h at RT. Purified antibodies 4D06 and 4D08 were serially diluted in PBS from 1000 nM to 0.03 nM and incubated for 2h at RT. After washing, the HRP-conjugated detection antibodies goat anti-human IgG (1:1000 in PBS; Sigma Aldrich) was added and incubated for 1h at RT. Finally, the assay was developed with TMB, and the reaction was stopped after 5’ upon adding 2N H_2_SO_4_. Absorbance was measured at 450 nm in a Tecan reader (Tecan Group AG). EC_50_ values were calculated by non-linear regression with GraphPad Prism.

For the analysis of different genotypes, pre-coated Murex HBsAg Version 3 ELISA stripes (DiaSorin) were activated as described by the manufacturer. 15 HBsAg-positive human plasma samples containing different HBV sub-/genotypes were added at 1 IU/well (Paul-Ehrlich-Institute, 1^st^ WHO International Reference Panel for HBV Genotypes for HBsAg Assays). Human serum from a patient tested negative for HBsAg was used as a negative control. After incubation for 1h at 37°C, biotinylated antibodies 4D06 and 4D08 were added at 2 µg/mL in PBS and further incubated for 30’ at 37°C. Biotinylation was performed in-house using an EZ-Link Sulfo-NHS-Biotinylation kit (Thermo Fisher). Avidin-HRP and TMB substrate were added for detection as described above.

### 2.6 HBV uptake inhibition

The capability of mAbs 4D06 and 4D08 to prevent HBV uptake was tested *in vitro* using HepG2 cells stably expressing human NTCP (HepG2-NTCP) (19). For this, cells were seeded in collagen-coated 24-well plates with a density of 3x10^5^ cells/well in DMEM differentiation medium (DMEM diff, i.e. 10% FCS, 1% penicillin-streptomycin, 1% glutamine, 1% NEAA, supplemented with 2.5% DMSO). Cells were differentiated for 3 days before infection. On the day of infection, 100 nM of 4D06 and 4D08 mAbs were pre-incubated with purified HBV with an MOI 100 for 30’ at 37°C in DMEM diff medium. Heparin (Ratiopharm) and HBIg (0.3 IU/well; Nabi-HB, Biotest Pharmaceuticals Corporation) were handled similarly and served as controls. Cells were pre-chilled on ice for 15’ before inoculation. The inoculum with HBV and antibodies was incubated for 1h, enabling the virus to bind to the cell surface. After incubation, cells were washed 2x with PBS, trypsinized for 3’, and shifted back to 37°C for 3-24h. The total cellular DNA was extracted from cell lysate using a NucleoSpin tissue kit (Macherey-Nagel) to determine intracellular HBV cccDNA as markers of HBV uptake. DNA was frozen at -20°C until cccDNA was quantified by qPCR, as previously described (19).

### 2.7 Analysis of antibody neutralization capacity

HepG2-NTCP cells were seeded with 3x10^5^ cells/well in collagen-coated 24-well plates in DMEM diff medium and differentiated for 3 days before infection. Purified HBV (MOI 100) was pre-incubated with serial dilutions of mAbs 4D06 and 4D08 from 0.01 nM to 1000 nM for 3h at 37°C. PEG6000 was added to the inoculum at a final concentration of 4% (v/v). The inoculum was incubated for 18h before. Cells were then washed 3x with PBS, and a fresh DMEM diff medium was added. To monitor the infection, the supernatant was collected for HBeAg measurement at days 4 and 8 post-infection. The experiment was terminated at day 8 post-infection when cells were lysed, and total DNA was extracted with the NucleoSpin tissue kit supplied by Macherey-Nagel. Intracellular cccDNA was quantified by qPCR as previously described (19). HBeAg was determined by commercial immunoassay (Siemens Molecular Diagnostics).

### 2.8 HDV neutralization assay

Hepatitis Delta virus (HDV) was enveloped with HBV envelope proteins of different HBV genotypes by co-transfection of Huh7 cells with pJC126 (HDV genotype 1, kindly provided by John Taylor) and the corresponding plasmids pLX304-HB2.7 coding for HBV genotypes A-H, respectively. The cell supernatant containing HDV carrying the different HBV envelopes was harvested from day 10-13 post transfection. For infection neutralization, Huh7-NTCP cells were seeded in 96-well plates the day before infection. On the day of infection, antibodies 4D06 and 4D08 were incubated with HDV-containing supernatant for 60’ at 37°C, then diluted 1:2 with medium containing 8% PEG and used as the inoculum for Huh7-NTCP cells. One day after infection, cells were washed and further cultivated. Cell culture medium was changed every 3 days. Cells were fixed with 4% PFA and analyzed for successful infection by immunofluorescence microscopy using an anti-HDAg antibody at day 7 post infection.

### 2.9 Retroviral transduction of T cells

T cells were enriched and stimulated using human T activator CD3/CD28 Dynabeads (Thermo Fisher) for 2 days in T cell medium with FBS: RPMI, 10% FBS, 1% pen/strep, 1% glutamine, 1% sodium pyruvate, 1% NEAA, 10 mM HEPES, 16.6 μg/ml Gentamycin (all Thermo Fisher), supplemented with 300 U/ml IL2. 0.45 µm-filtered retrovirus cell culture supernatant from stable producer cell lines was centrifuged at 2000xg, 32°C for 2h on non-tissue culture-treated plates (Corning) coated with 20 µg/ml RetroNectin for 2h (Takara). Retrovirus cell culture supernatant was removed, and T cells were spinoculated onto the virus-coated plate at 1000xg for 10’. A 2^nd^ transduction was performed after 24h. CAR expression was determined by flow cytometry with anti-human CD4-APC (eBioscience), anti-human CD8-PB (BioLegend), and anti-HA-PE (BioLegend), diluted in PBS with 0.1% BSA (Sigma-Aldrich). Cells were analyzed using a CytoFLEX S (Beckman Coulter), and data were analyzed with FlowJo 10.4 software.

### 2.10 Co-cultures on plate-bound antigen

HBsAg was coated on cell culture 96-well plates ON at 37°C in concentrations from 0.1-10 µg/ml. Plates were washed 2x with ELISA, and CAR-transduced T cells (CD4^+^ to CD8^+^ ratio ~1:1) were added with 1x10^5^ transduced cells/well. IFNγ in the cell culture supernatant was detected after 48h using an ELISA kit (Thermo Fisher), according to the manufacturer’s instructions. EC_50_ values were calculated by non-linear regression with GraphPad Prism.

### 2.11 Co-cultures of CAR-T cells with HepG2-derived target cells

HepG2-derived cell lines were seeded in DMEM diff medium on collagen-coated plates, and the medium was changed every 2-3 days for 10-12 days. For coculture, T cells (CD4^+^ to CD8^+^ ratio ~1:1) were added in equal amounts of T cell medium (final concentration of 1% DMSO in coculture) at different effector-to-target ratios. IFNγ in the cell culture supernatant was detected using an ELISA kit (Thermo Fisher) according to the manufacturer’s instructions. For intracellular cytokine staining, 2 µg/ml Brefeldin A (Sigma-Aldrich) was added 16h after co-culture start and staining was performed after 48h, using the Fixation/Permeabilization kit (BD), live/dead Fixable Aqua stain (Thermo Fisher), anti-human CD4-PerCP (BioLegend), anti-human CD8-FITC (Thermo Fisher), anti-HA-PE (BioLegend), anti-human IFNγ-AF700 (BD Biosciences), anti-human TNFα-APC (BioLegend), anti-human IL2-PE-Cy7 (Thermo Fisher), anti-human GrzB-PB (Thermo Fisher). CellTrace Violet Stain (Thermo Fisher) was used for proliferation analysis according to the manufacturer’s instructions.

### 2.12 Real-time cytotoxicity measurement

HepG2-derived cell lines were differentiated in cell culture flasks with a change of DMEM diff every 2-3 days for 7-9 days. Cells were then seeded onto 96-well electronic microtiter plates (ACEA Biosciences) with 5x10^4^/well and differentiated for three additional days. CAR-transduced T cells (CD4^+^ to CD8^+^ ratio ~1:1) were added at different effector-to-target ratios. Electrical impedance was measured every 30’ with an xCELLigence SP real-time cell analyzer (ACEA Biosciences).

### 2.13 CAR-T cell treatment of AAV-HBV infected mice

Homozygous B6.129S7-Rag1^tm1Mom^ (Bl6.Rag 1^-^/^-^) mice were bred in house in a specific pathogen-free animal facility. Persistent HBV replication was established by intravenous injection of 1.8×10^10^ genome equivalents of an adeno-associated virus vector containing a replication competent 1.3-fold HBV genome (AAV-HBV) (20). T cells were isolated from CD45.1^+/+^ C57BL/6 donor mice and retrovirally transduced with the CAR constructs as described previously (11). 5 weeks after AAV-HBV infection, 1x10^6^ CAR- or mock-transduced T cells per mouse were transferred by i.p. injection. HBsAg, HBeAg and ALT levels as well as intrahepatic pgRNA were quantified as described previously (21).

### 2.14 Restimulation and cytokine secretion of mouse cells from blood, spleen and liver

Blood cells, splenocytes and liver-associated lymphocytes were isolated as described previously (21). For intracellular cytokine staining, cells were stimulated for 16h in the presence of 1 µg/ml Brefeldin A (Sigma-Aldrich) with plate-bound HBsAg at 10µg/ml. Surface staining was performed using anti-CD8-PB (BD), anti-CD4-AF700 (BioLegend) and anti-HA-PE (BioLegend) antibodies. Dead cells were excluded from analysis with live/dead Fixable Aqua stain (Thermo Fisher). Intracellular cytokine staining was performed using a Fixation/Permeabilization kit (BD) according to the manufacturer’s instructions with anti-IFNγ-FITC (BD) and anti-TNFα-PE-Cy7 (BD) antibodies. Cells were analyzed using a CytoFLEX S (Beckman Coulter), and data were analyzed with FlowJo 10.4 software.

### 2.15 Immunohistochemistry

Liver, kidney and heart tissue samples were fixed in 4% buffered formalin for 24h and were paraffin embedded. Tissue sections that were 2-μm-thin were then prepared with a rotary microtome (HM355S, Thermo Fisher, Waltham, USA). Immunohistochemistry was performed using a Bond RX system (Leica, Wetzlar, Germany) with the anti-HBcAg primary antibody (LSBio, LS-C312204, 1:50 dilution) and a horseradish peroxide coupled secondary antibody. Briefly, the slides were deparaffinized using deparaffinization solution pre-treated with epitope retrieval solution 2 (corresponding to EDTA buffer pH9) for 40’. Antibody binding was detected with a polymer refine detection kit without post primary reagent and was visualized with DAB as a dark brown precipitate. Counterstaining was done with haematoxylin eosin. Slides were scanned using an Aperio AT2 slide scanner (Leica, Wetzlar, Germany). HBcAg-positive hepatocytes were determined based on the localization, intensity, and distribution of the signal in 10 random view fields (8× magnification). The mean numbers of the HBcAg-positive hepatocytes were quantified per mm^2^.

### 2.16 Statistical analysis

Statistical analyses were done with the Prism 10.1.2 software. Statistical differences were calculated using student t-test. P-values ≤0.05 were considered significant.

## 3 Results

### 3.1 Isolation of HBsAg-specific memory B cells with ELISpot and flow cytometry

For the generation of monoclonal antibodies from the peripheral blood of an immune donor, we used single B-cell sorting technology (16–18). This approach relies on the frequency of circulating antigen-specific memory B cells, which is generally extremely low and highly dependent on the immune status of a person (22–24). Thus, identifying and isolating those rare antigen-specific B cells is challenging and depends on a suitable donor. In addition, an optimized sorting strategy is also required to separate the specific signal of low-frequency B cells from background staining.

**Figure 1:**
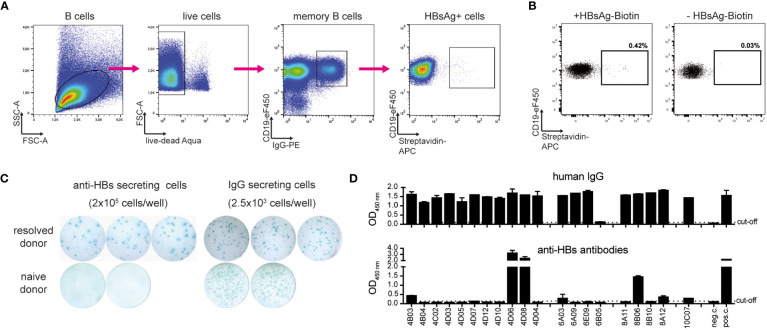
Identification and isolation of HBV-specific memory B cells. **(A)** Representative gating strategy to identify and isolate live CD19^+^ IgG^+^ HBsAg^+^ memory B cells of a donor with resolved acute HBV infection after magnetic B-cell enrichment and incubation with HBsAg-biotin. Arrows indicate stepwise gating. **(B)** HBsAg^+^ gate of B cells of the donor with resolved acute infection. Comparison to a staining control using streptavidin-APC. Depicted cell populations were pre-gated on live CD19^+^ IgG^+^ B cells. Frequencies refer to IgG^+^ cells. **(C)** Representative ELISpot well images of HBsAg-specific and total IgG. ELISpot was performed after *in vitro* stimulation of PBMC for five days. **(D)** Transfection of HEK293 cells with corresponding antibody heavy and light chain plasmids. Secretion of total IgG (detected with polyclonal goat ant-human IgG HRP) and anti-HBs-specific antibodies (on protein G-coated plates, detected with HBsAg-biotin and Avidin-HRP) was determined via ELISA. Medium only served as negative control (neg. c.). Polyclonal HBIg diluted in medium served as a positive control (pos. c.). Data points represent mean values ± SD from triplicates.

To identify low-frequency HBV-specific human memory B cells from peripheral blood, HBsAg was purified from human serum and labeled with biotin. The resulting HBsAg-biotin was used as a decoy to selectively bind to HBsAg-specific B-cell receptors on the surface of IgG^+^ CD19^+^ human memory B cells. B cells were enriched from PBMC by negative selection to decrease unspecific binding of HBsAg-biotin to non-B cells. HBsAg-specific memory B cells were identified through incubation with HBsAg-biotin and fluorophore-coupled streptavidin via a four-step gating strategy (**Figure 1A**) from a donor with a high serum anti-HBs titer who had previously resolved an acute HBV infection and observed a frequency of 0.42% HBsAg^+^ CD19^+^ IgG^+^ B cells out of the total IgG^+^ cells (**Figure 1B**). Incubation with streptavidin only served as a control for non-specific binding to B-cell receptors and indicated background binding of 0.03% of IgG^+^ B cells. Staining of an HBV-naïve donor sample showed background frequencies of 0.15% and 0.04%, respectively (**Supplementary Figure 1**). Considering that IgG^+^ B cells comprise approximately 15% of the B-cell population (25), the frequency of HBsAg-specific B cells of CD19^+^ B cells was calculated with 0.06% for the donor with resolved acute HBV infection.

To further verify the frequency of antigen-specific B cells obtained by flow cytometry, an HBsAg-specific B cell ELISpot was performed in parallel. PBMC were stimulated *in vitro* for five days with IL2 and the immune adjuvant R848 to induce the differentiation of memory B cells into antibody-secreting cells. After transferring the cells to IgG- and HBsAg-coated ELISpot plates, antibody secretion was determined by spot counting. While total IgG secretion was high both for the donor with resolved HBV infection as well as a naïve donor sample which served as negative control, anti-HBs antibodies were secreted only by cells from the donor with resolved infection with frequencies ranging at approximately 0.01% (20 spots per 2.0x10^5^ cells) of HBsAg^+^ cells in PBMC (**Figure 1C**). Our numbers agree with previous publications using a similar method for identifying HBV-specific B cells and convinced us to continue with B-cell isolation (26, 27).

After successfully evaluating HBsAg-biotin as a specific decoy to capture HBsAg-reactive memory B cells, single-cell sorting of those cells was performed. B cells were enriched from the PMBC of the donor with resolved infection and incubated with HBsAg-biotin. CD19^+^ IgG^+^ and HBsAg^+^ memory B cells were single-cell-sorted based on the gating strategy described above (**Figure 1A**). 238 antigen-positive single cells were sorted into 96-well PCR plates containing lysis buffer and frozen immediately on dry ice.

### 3.2 Identification of human immunoglobulin variable chain genes and cloning of full-length, recombinant anti-HBs antibodies

To generate recombinant human monoclonal antibodies, mRNA was isolated from each single-sorted HBsAg^+^ memory B cell and reverse transcribed into cDNA. Human immunoglobulin variable heavy, kappa light, and lambda light chain genes were recovered using nested PCR according to the protocol published by *Tiller et al*. (16). While the overall amplification efficacy was low, corresponding PCR products of heavy and light chain genes were obtained for 27 single-sorted B cells, which were then purified and sequenced. Most PCR products represented productive rearranged Ig entities, and germline V(D)J-gene segments with the highest identity were identified using the international immunogenetics information system (IMGT) (28). Based on their specific sequence, corresponding variable heavy and light chain couples were cloned as recombinant, full-length IgG1 antibodies, and 20 clones were expressed in HEK293 cells. Most constructs showed a high secretion of total IgG. Two antibodies, namely 4D06 and 4D08 that gave the strongest anti-HBs signal (**Figure 1D**) were selected for further analysis. 4D06 and 4D08 were then re-expressed in HEK293 cells and purified by protein G column affinity purification to a purity of >95% (**Supplementary Figure 2**).

### 3.3 4D06 and 4D08 mAbs recognize a conformational versus a linear epitope of the HBV S-protein

**Figure 2:**
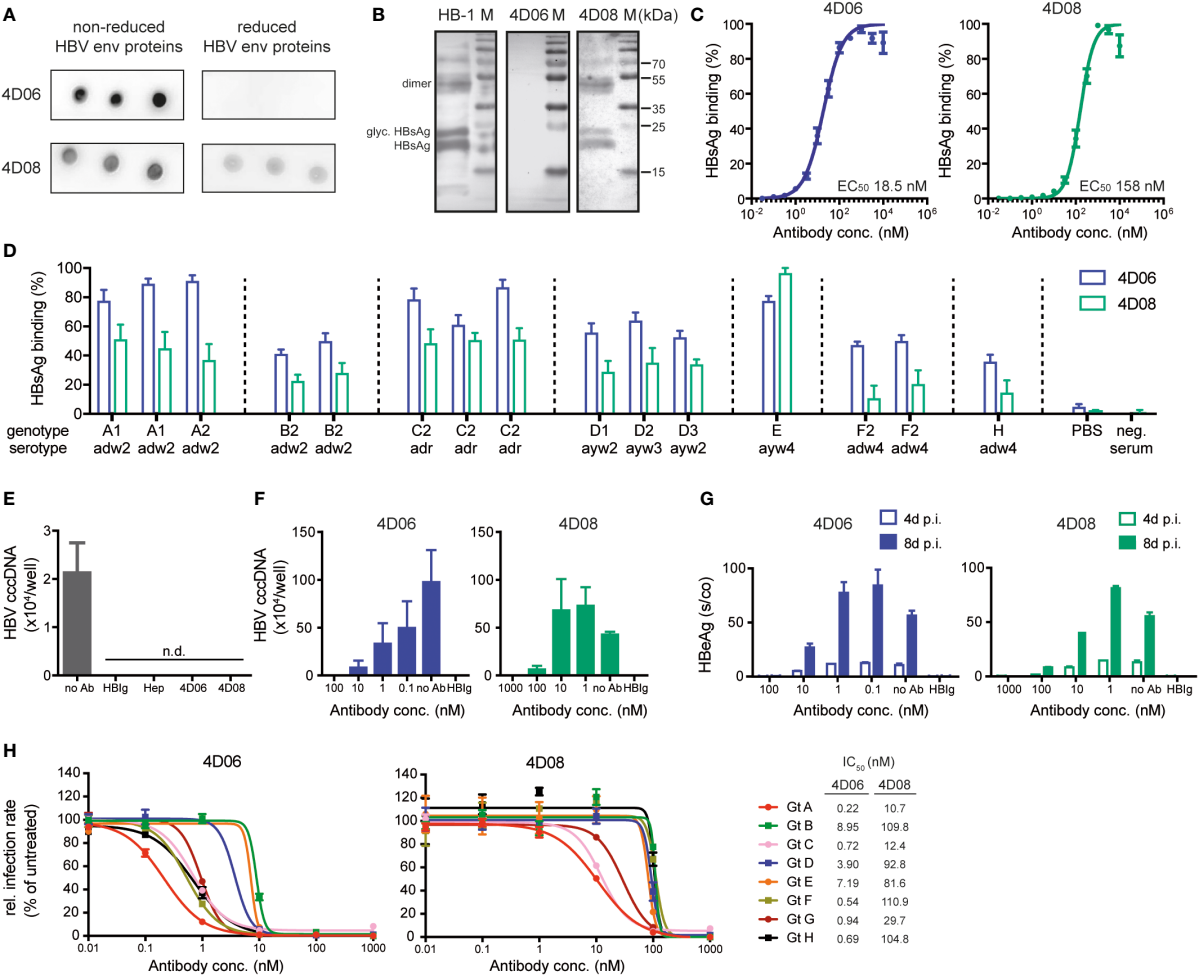
Characterization of mAbs 4D06 and 4D08. **(A)** Dot blot analysis of the interaction between mAbs and HBV envelope (HBV env) proteins blotted on a PVDF membrane under reducing or non-reducing conditions. 4D06 and 4D08 served as primary antibodies, goat anti-human IgG HRP was used for detection. **(B)** Western blot analysis of the interaction of HBV env proteins with mAbs. HBV env proteins were separated by SDS PAGE under reducing conditions and blotted on a PVDF membrane. Env protein detection was performed as described in **(A)**. Bands represent non-glycosylated and glycosylated (glyc.) forms of monomeric HBsAg and dimeric (dimer) HBsAg. HB-1 served as a positive control for an antibody recognizing a linear epitope. **(C)** ELISA analysis of the interaction between mAbs and plate-bound HBsAg; polyclonal goat anti-human IgG HRP antibody was used for detection. EC_50_ values were calculated by nonlinear regression. **(D)** ELISA analysis for the interaction between mAbs and HBsAg from several HBV geno- and serotypes, based on 15 HBsAg-positive human plasma samples from the Paul-Ehrlich Institute (Langen, Germany). HBsAg was captured with HBsAg-specific antibodies from a commercial anti-HBs ELISA kit (DiaSorin, Italy). The binding of biotinylated mAbs 4D06 and 4D08 was detected with Avidin-HRP. Data points are normalized to the highest value. **(E)** HBV uptake assay with differentiated HepG2-NTCP cells inoculated with HBV after pre-incubation with heparin (Hep), HBIg (0.3 IU/well), 4D06 or 4D08 (100 nM). No Ab served as neg. control. Absolute quantification of intracellular HBV cccDNA 24 hours post-infection (p.i.). n.d. = not detectable. **(F)** Neutralization assay: Antibodies were pre-incubated with HBV, followed by inoculation of HepG2-NTCP cells with HBV. Absolute quantification of intracellular HBV cccDNA eight days p.i.; IC_50_ determination with non-linear regression. **(G)** Level of HBeAg in the cell culture supernatant four and eight days p.i. **(H)** Huh7-NTCP cells were preincubated with 4D06 or 4D08 and inoculated with HDV, enveloped with HBV proteins of genotypes (Gt) A-H. Cells were analyzed for HDAg by immunofluorescence assay 7 days p.i. IC_50_ concentration of 4D06 and 4D08 were calculated with non-linear regression. All data points represent mean values ± SD from triplicates.

A dot blot was performed to evaluate whether 4D06 and 4D08 recognize a linear or a conformational epitope within the HBV env protein. Recombinant HBsAg was dotted onto a membrane either under non-reducing conditions, keeping the native three-dimensional structure of HBsAg, or under reducing conditions, leading to protein linearization. Both mAbs 4D06 and 4D08 recognized the dotted antigen under native conditions; however, the reactivity was lost for 4D06 under reducing conditions, while it remained intact for 4D08. This strongly indicates that 4D08 recognizes a linear epitope while 4D06 recognizes a conformational epitope, since a loss of conformational integrity of an epitope is expected under the reducing assay conditions (**Figure 2A**). A Western blot of denatured HBV-positive cell lysates separated by SDS-PAGE was performed to verify this observation. Two bands at 27 kDa and 24 kDa were detected with 4D08 and HB-1 antibodies, confirming that the antibodies recognize glycosylated and non-glycosylated HBsAg. The HB-1 control antibody recognizes a known linear epitope in the small HBV env protein at position S119-125 in the antigenic loop (29). Additionally, one band with a molecular weight of approximately 50 kDa was detected, most likely representing a dimer of the S protein (30) (**Figure 2B**). In conclusion, 4D06 recognizes a conformational epitope, whereas 4D08 binds a linear epitope on the HBVenv proteins.

### 3.4 4D06 and 4D08 are high-affinity antibodies with broad neutralization capacity

Antibody affinities were determined by ELISA using serial dilutions of mAbs 4D06 and 4D08 on plate-bound HBsAg. The calculation of half-maximal effective concentrations (EC_50_) via non-linear regression revealed high affinities of both antibodies in the nanomolar range, i.e. 18.5 nM for 4D06 and 158 nM for 4D08 (**Figure 2C**). Next, we investigated the reactivity of 4D06 and 4D08 against the eight major HBV genotypes (A-H), five of which (genotypes A, B, C, D, and E) are responsible for causing 96% of chronic HBV infections worldwide (31). An indirect ELISA using a WHO genotype reference panel for HBsAg assays from the Paul-Ehrlich Institute (Langen, Germany) showed a broad reactivity of 4D06 and 4D08 towards all tested genotypes and serotypes *adw*, *ayw*, and *adr*, with 4D06 slightly outperforming 4D08 on most genotypes (**Figure 2D**).

We then analyzed the capacity of 4D06 and 4D08 to prevent virus uptake *in vitro*. Differentiated HepG2-NTCP cells (19), susceptible to HBV infection, were used as target cells and pre-incubated with mAbs 4D06 and 4D08 or polyclonal anti-HBs antiserum (HBIg) or heparin, both of which are known to inhibit HBV uptake (32) and served as positive controls. Target cells were then inoculated with HBV at 4°C, allowing virus attachment, before shifting to 37°C to induce synchronized HBV uptake (33). 24 hours after inoculation, no nuclear transcription template of HBV, the so-called covalently closed circular DNA (cccDNA), was detected via qPCR, indicating successful infection prevention by pre-incubation with 4D06 and 4D08 (**Figure 2E**).

Last, the neutralization capacity of both antibodies was quantified using the mAbs at increasing concentrations. The establishment of an HBV infection was quantified through cccDNA qPCR in cell lysates at day 8, where a superior neutralization capacity was observed for 4D06 compared to 4D08 (**Figure 2F**). The measurement of secreted HBeAg at days 4 and 8 post-infection showed a concentration- and time-dependent increase of HBeAg, with 4D06 achieving complete neutralization at a concentration of 100 nM, while 4D08 only reached a comparable result at 1000 nM (**Figure 2G**). To evaluate the infection neutralization capacity of 4D06 and 4D08 mAbs against the full range of HBV genotypes, we made use of the fact that HDV uses the same envelope as HBV. HDV was enveloped with HBV envelope proteins from all genotypes A to H and used for infection neutralization with 4D06 and 4D08 at increasing concentrations to determine the 50% inhibitory concentration (IC_50_) (**Figure 2H**). This confirmed that 4D06 and 4D08 are high-affinity antibodies that broadly neutralize different HBV geno- and serotypes.

### 3.5 CAR constructs derived from 4D06 and 4D08 mAbs are functional in primary human T cells

**Figure 3:**
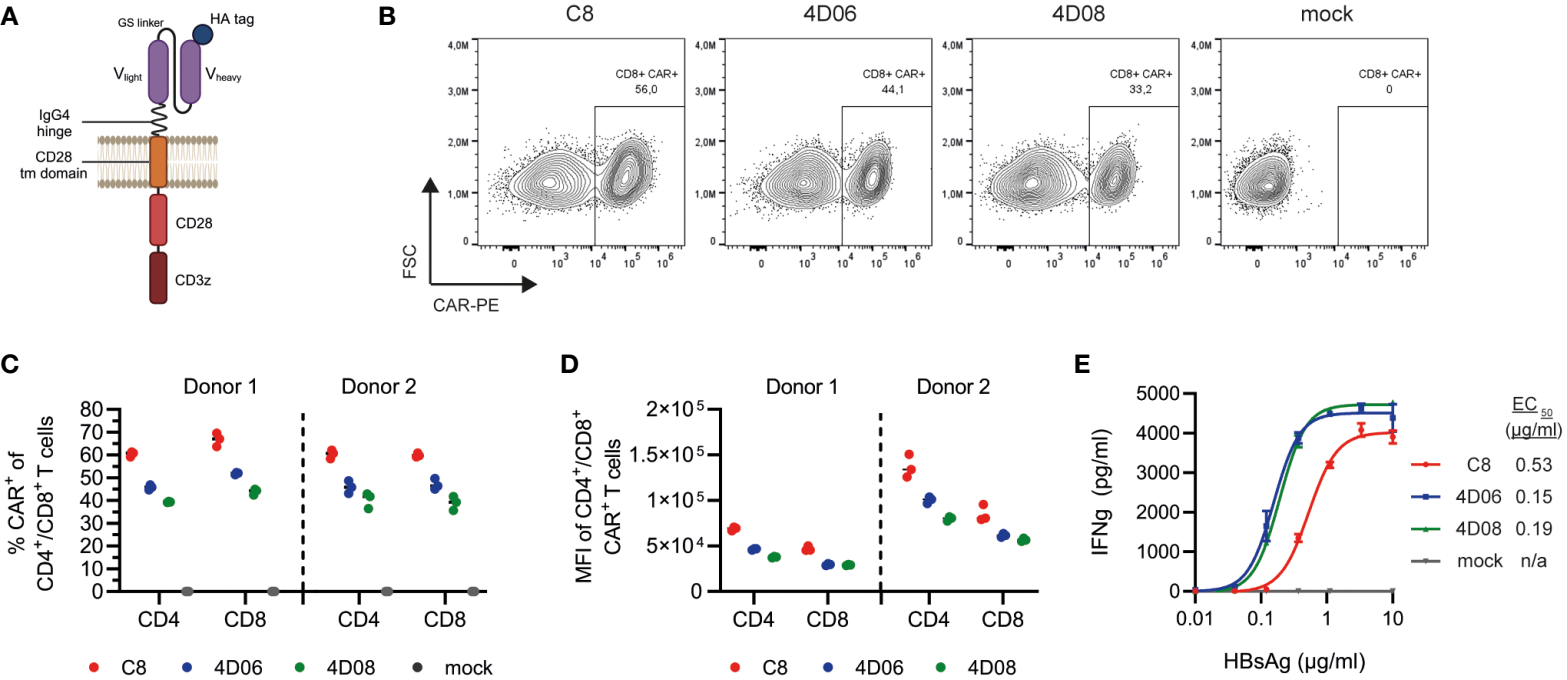
Expression and functional characterization of CARs using different S-binders. **(A)** Schematic representation of CAR constructs. **(B)** Exemplary flow cytometry plots of retroviral transduction of primary human CD8^+^ T cells with C8-, 4D06-, and 4D08-CARs. **(C)** Transduction rates of CD4^+^ and CD8^+^ T cells from two healthy donors for C8-, 4D06-, and 4D08-CARs determined by flow cytometry. **(D)** Expression levels of C8-, 4D06-, and 4D08-CARs shown as mean fluorescence intensity (MFI) of CAR+ population (does not apply to mock cells). **(E)** 1x10^5^ CAR-transduced T cells were cultured on plate-bound HBsAg titrated from 0.01-10 µg/ml. IFNγ secretion was measured after 48h by ELISA. Data points represent mean values ± SD from triplicates.

To explore the potential of our novel mAbs 4D06 and 4D08 for adoptive T-cell therapy, we constructed 2^nd^ generation CARs composed of an antibody-binding domain with an HA-tag, an IgG4 hinge region, a CD28 transmembrane domain, and intracellular signaling moieties of CD28 and CD3zeta (**Figure 3A**). The respective binding domain of each CAR construct consisted of a scFv using a glycine-serine linker connecting codon-optimized variable heavy and light chains of either the 4D06- or the 4D08-antibody. We used the C8-binder from an HBV-specific S-CAR previously generated in our lab (14) as a control. Primary human T cells were successfully grafted with all three CARs through retroviral transduction (**Figure 3B**). All CAR constructs expressed well in both CD4^+^ and CD8^+^ T cells from healthy donors, with the C8-CAR consistently generating higher transduction rates than the 4D06- and 4D08-CARs (**Figure 3C**). A higher expression level of the C8-CAR than the 4D06- and 4D08-CARs was confirmed by considering the mean fluorescence intensity of flow cytometry staining (**Figure 3D**).

CAR-transduced T cells were cultured on titrated plate-bound HBsAg to compare the novel CAR constructs to the existing C8-CAR regarding their binding avidity. Unlike their mAb counterparts, 4D06- and 4D08-CARs showed very comparable EC_50_ values with 0.15 and 0.19 µg/ml, whereas the C8-CAR avidity ranged approximately three times lower with an EC_50_ of 0.53 µg/ml (**Figure 3E**). In summary, all three CAR constructs were functional in primary human T cells and showed avidities in the nanomolar range, with the new 4D06- and 4D08-CARs outperforming the pre-existing C8-CAR in terms of sensitivity at lower antigen levels.

### 3.6 4D06- and 4D08-CARs are polyfunctional and able to kill HBV-expressing target cells

To further characterize the functionality of the new constructs, we performed co-culture experiments of CAR-transduced T cells with a set of hepatoma cell lines: HepG2 cells representing the non-HBV-expressing control; HepG2-SML cells expressing the HBVenv proteins S, M and L under the endogenous HBV promotors at high levels; HepG2-2.15 cells carrying four copies of a dimer HBV genome as integrates and expressing HBV-protein at a lower level.

**Figure 4:**
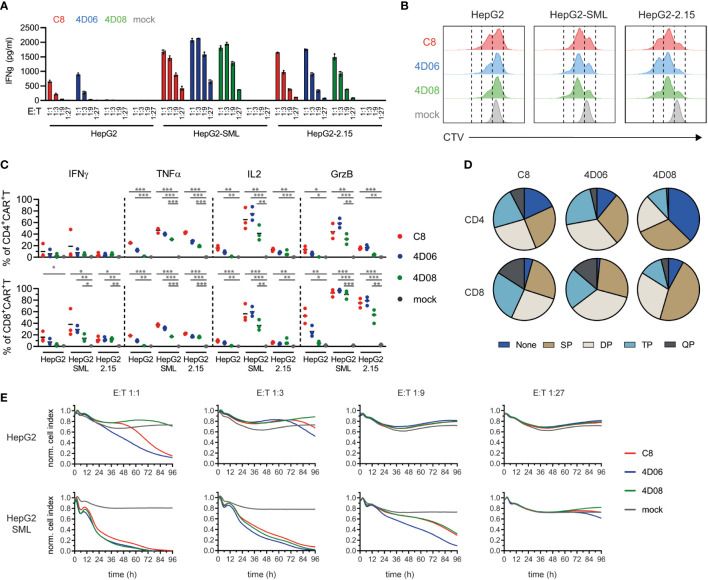
Functional evaluation of CARs from novel binders on HBsAg-expressing target cells. **(A)** CAR-transduced T cells were co-cultured at indicated effector-to-target (E:T) ratios on HepG2, HepG2-SML, or HepG2-2.15 cells. IFNγ secretion was measured via ELISA after 72h. Data points represent mean values ± SD from triplicates. **(B)** CAR-transduced T cells were pre-incubated with CellTrace Violet (CTV) and cultured at an E:T ratio of 2:1 on indicated target cells. Proliferation as a measure of CTV decrease was observed by flow cytometry after 72h. Histograms are representatives of duplicates. **(C)** Intracellular cytokine staining of CAR-transduced T cells co-cultured at an E:T ratio of 2:1 for 48h on indicated target cells (2 µg/ml Brefeldin A added after 24h). Statistical differences were calculated using unpaired student t-test; *=p≤0.05, **=p≤0.01, ***=p≤0.001. **(D)** Polyfunctionality of CAR-transduced T cells derived from intracellular cytokine staining described in **(C)**, indicating single (SP), double (DP), triple (TP), or quadruple positive (QP) populations of CD4^+^ or CD8^+^ CAR-transduced T cells incubated on HepG2-SML cells. **(E)** xCELLigence cytotoxicity assay of the different CAR-transduced T cells co-cultured at various E:T ratios for 96h with the indicated target cells. Data points represent mean values from triplicate analyses.

On HBV-expressing target cells, cytokine secretion from T cells grafted with all three CARs was comparable and strongly depended on the effector-to-target (E:T) ratio (**Figure 4A**). However, we observed that CAR constructs C8 and 4D06 induced IFNγ-secretion not only on HBV-expressing target cells but also on HepG2 control cells at 1:1 and 1:3 E:T ratios. This effect is most likely due to tonic signaling of these two CAR-constructs. The 4D08-CAR, in contrast, showed no such activity (**Figure 4A**). On HBV-expressing target cells, however, cytokine secretion from T cells grafted with all three CARs was comparable and strongly depended on the E:T ratio (**Figure 4A**). Cell trace violet staining in flow cytometry showed a marked proliferation of T cells grafted with all three CAR constructs when exposed to their cognate antigen on HepG2-SML and on HepG2-2.15 cells. On HepG2-2.15 cells, T cells grafted with the 4D06-CAR slightly outperformed those grafted with the C8-CAR, followed by the 4D08-CAR (**Figure 4B**). However, C8- and 4D06-CAR T cells also exhibited a pronounced, antigen-independent proliferation on HepG2 control cells. Interestingly, this again was lower for 4D08-CAR T cells. All three constructs induced the secretion of varying amounts of cytokines as observed through intracellular staining in flow cytometry, with CD4^+^ T cells generally secreting higher amounts of TNFα and IL2 and CD8^+^ T cells secreting more IFNγ and GrzB (**Figure 4C**). Once again, C8- and 4D06-CARs displayed a distinct background activation on HepG2 cells compared to the 4D08-CAR, which was not activated in the absence of antigen. The C8- and 4D06 CARs also secreted more cytokines than the 4D08-CAR when co-cultivated with HepG2-SML and HepG2-2.15 cells.

Interestingly, when looking at the polyfunctionality of T cells on HepG2-SML cells, it appeared that C8- and 4D06-transduced CAR T cells shared a rather similar profile with a substantial subset of triple or quadruple positive cells among both CD4^+^ and CD8^+^ T cells, whereas 4D08-transduced T cells seemed to have a much higher proportion of single positive cells or cells that didn’t secrete any of the cytokines tested, in particular in the CD4^+^ subset (**Figure 4D**). Last but not least, CAR-transduced T cells were able to effectively kill HBsAg-expressing target cells, as shown by an xCELLigence cytotoxicity assay (**Figure 4E**). C8-CAR and 4D06-CAR T cells once again showed background activation and cytotoxicity on HepG2 control cells, particularly at an E:T ratio of 1:1, which was absent for the 4D08-CAR. Remarkably, on HepG2-SML cells, all three CAR constructs performed similarly well regarding target cell killing at E:T ratios 1:1 and 1:3 (**Figure 4E**). This also held true for HepG2-2.15 cells that present much lower levels of HBVenv proteins (Supplementary **Figure 3**).

In conclusion, T cells transduced with all CAR constructs show a polyfunctional cytokine secretion profile upon antigen binding. However, it seems that the 4D08-CAR shows an overall diminished background activation compared to C8- and 4D06-CARs, which concurs with a different cytokine secretion profile. At the same time, the killing capacity towards HBV-expressing target cells in cell culture remained similar for all three constructs.

### 3.7 4D06- and 4D08-CARs are safe and functional *in vivo*

**Figure 5:**
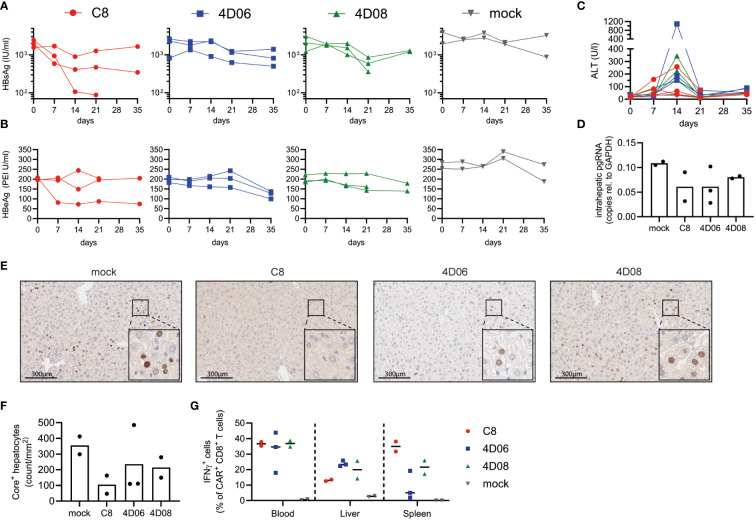
*In vivo* evaluation of CARs from novel binders in murine model of chronic HBV infection. Persistent HBV replication was established in Bl6.Rag1^-^/^-^ mice using AAV-HBV. 1x10^6^ C8-, 4D06- or 4D08-CAR CD45.1^+/+^ syngeneic T cells per mouse were transferred at week 5 after AAV-HBV infection (n=3). Two animals served as mock control. End-point analyses were performed at day 21 or 35 as indicated. Kinetics of **(A)** HBsAg, **(B)** HBeAg and **(C)** ALT levels in serum are given. **(D)** HBV pregenomic RNA (pgRNA) was quantified in liver tissue lysates obtained at day 35 through RT-PCR. **(E)** Immunohistochemistry of HBcAg expression in the liver of one representative animal per treatment group. Visible dark brown precipitates in the nucleus indicate HBV-positive hepatocytes. Scale bar represents 300 μm. **(F)** Number of HBcAg^+^ hepatocytes per mm^2^ as determined by quantitative analysis of immunohistochemistry stainings. **(G)** Frequencies of IFNγ-secreting CAR^+^ CD8^+^ T cells from blood, liver or spleen detected by intracellular cytokine staining after 16h *ex vivo* stimulation with plate-bound HBsAg. Symbols and lines represent individual mice.

Finally, we tested our constructs in an initial proof-of-concept experiment in immunodeficient Bl6.Rag1^-^/^-^ mice infected with AAV-HBV to model chronic HBV infection. Bl6.Rag1^-/-^ mice were chosen to avoid an immune response against the fully human CARs (11). Upon transfer of 1x10^6^ CAR-T or mock cells per mouse, serum levels of the viral HBsAg (**Figure 5A**) and HBeAg (**Figure 5B**) declined over time in mice treated with all three constructs, with the most significant decrease observed for C8-CAR-treated mice. As expected, the decline of circulating antigens was slow, because the immunodeficient mice cannot raise an antibody response contributing to HBV clearance. CAR-T cells caused moderate but transient liver damage, with serum ALT levels peaking at day 14 and normalizing again thereafter (**Figure 5C**). A decline of intrahepatic pregenomic HBV RNA in CAR-T-treated groups indicated a notable effect on HBV replication (**Figure 5D**). In line with diminished HBV replication, immune histochemistry staining of liver tissue samples showed a drop in HBcAg expression in the mice of all CAR-T-treated groups (exemplary mice **Figure 5E**, quantitative analysis **Figure 5F**). Haematoxylin eosin staining revealed some lymphocyte infiltration, but no significant tissue damage in liver, heart or kidney of the mice (**Supplementary Figure 4**). Upon restimulation with HBsAg, lymphocytes isolated from blood, liver and spleen secreted higher amounts of IFNγ (**Figure 5G**) and TNFα (**Supplementary Figure 5**) in CAR-T-treated mice compared to mock animals. In summary, all CAR constructs appear to be safe and functional in a mouse model of chronic HBV infection.

## 4 Discussion

In this study, we describe a fast and efficient method to identify fully human HBV-specific monoclonal antibodies from donors with resolved infection using single B-cell sorting technology, which is subsequently used to generate chimeric antigen receptors for the use in adoptive T-cell therapy of hepatitis B virus infection or HBV-induced hepatocellular carcinoma. To date, different approaches have been used for the generation of HBV-specific mAbs, mostly using classical hybridoma technology (29, 34–36), phage display technologies (37, 38) or immortalization of human peripheral blood B cells with Epstein-Barr virus (39, 40). However, these methods have drawbacks, such as the high probability of inducing harmful immune responses in humans against murine protein sequences or the rather inefficient process of B-cell immortalization (41). Single B-cell antibody technology represents a major improvement to the existing processes. With this approach, single antigen-specific human B cells can be isolated from small quantities of donor cells with an unbiased approach, and recombinant antibodies can be generated based on amplified immunoglobulin variable chain genes (16, 42, 43). It is straightforward and efficient and has already successfully led to the generation of monoclonal antibodies targeting the Dengue virus, human immunodeficiency virus type 1 (HIV-1), or Zika virus (24, 43–45).

Although the frequency of antigen-specific memory B cells in the blood of our donor was low, it was comparable with frequencies obtained by others ranging from 0% to 4% of CD19^+^ B cells (26, 27, 46). This convinced us to use our flow cytometry staining protocol for the isolation of single HBsAg-specific memory B cells for antibody generation, yielding a total of 20 novel human mAbs, with 4D06 and 4D08 showing the highest affinity for HBsAg. EC_50_ values were in a nanomolar range comparable to other previously published anti-HBs-reactive mAbs (29, 34, 47).

Detecting a particular epitope is important for the potential of monoclonal antibodies to neutralize HBV infection (48, 49). Most published neutralizing HBsAg-specific mAbs recognize conformational epitopes in the a-determinant of HBsAg, which is the most important antigenic region in the S-domain of the HBVenv proteins (29, 35, 47). Epitope characteristics of 4D06 and 4D08 varied: 4D06 only recognized the native protein, suggesting a conformational epitope formed by different amino acids across the protein. In contrast, 4D08 could recognize both the native and the reduced form of the small HBVenv protein, pointing towards a linear epitope. To further map both antibody epitopes, HBsAg mutants with site-directed mutations covering the extracellular domains of the small HBVenv protein would be required, similar to the analysis *Zhao et al*. performed for the C8-binder in a monoclonal antibody format (15).

Virus neutralization and uptake inhibition can efficiently prevent HBV infection. Both mAbs showed a concentration-dependent neutralization capacity and recognized various HBV geno- and serotypes. 4D06 was slightly superior to 4D08, as a 10-fold lower concentration was needed to achieve complete neutralization. This may be due to the recognition of a conformational epitope since it was reported that mAbs that recognize conformational epitopes are more potent in neutralization than antibodies that detect linear epitopes (37, 48).

Broad neutralization is an antibody property first described for monoclonal antibodies generated against HIV-1 (50). Broadly neutralizing antibodies are essential to treat HIV-1 as this virus is very diverse, and those mAbs can neutralize more than one strain (51). Broadly neutralizing antibodies recognize epitopes in conserved regions of HBsAg, which are less affected by amino acid substitutions introduced upon virus mutation but rather by the virus origin (29, 38). As HBV comes in nine genotypes (A-I) and four serotypes (*adw*, *ayw*, *adr*, *ayr*) (31), broadly neutralizing antibodies such as 4D06 and 4D08 are required to combat HBV around the globe. The observed differences between 4D06 and 4D08 mAb recognition of genotypes may be due to sequence variations in their respective epitopes, as different genotypes vary at least 7.5% across the complete genome (52).

The binding efficacy of anti-HBV antibodies and their capability to neutralize the virus has been reported to be highly dependent on the specific epitope recognized within the HBVenv protein (35) and antibodies recognizing a particular linear epitope on the small HBVenv protein were shown to be very potent against escape mutations (49). The higher affinity we observed for 4D06-mAb compared to 4D08-mAb correlated with a more substantial neutralization capacity in infection assays. However, this did not translate to an advantage in a CAR format, where avidities for both constructs were very similar and approximately 3-fold higher than the C8-CAR, despite its overall higher transduction rate and expression. According to a meta-study by *Mao et al.*, binders with moderate affinity may be preferred for the CAR format as they show less off-target toxicity and seem more effective (53). This was also discussed by *Jayaraman et al.*, who attributed a higher therapeutic window to CAR constructs with lower affinity targeting tumor antigens based on their enhanced ability to distinguish between high and low target expression on tumors versus on healthy tissue (54).

In addition, we observed lower unspecific activation for the 4D08-CAR. In our case, the CARs recognize non-self, virus-derived HBVenv antigens not expressed on HBV-negative control cells. This led us to conclude that the 4D06 and the C8-CAR activate constitutive signaling in T cells, known as tonic signaling. Such tonic signaling is critical to CAR specificity and CAR-T cell function as it predisposes the cells to exhaustion (55). Based on our data, in particular the distinct polyfunctionality profile of 4D08-CAR versus 4D06- and C8-CAR, we hypothesize that binding of a linear epitope could be one of the factors contributing to lower tonic signaling and may be beneficial for CAR functionality. Little is known about CAR constructs recognizing linear versus conformational epitopes. To further validate our hypothesis, we propose comparing a broader range of CAR constructs recognizing conformational or linear targets for HBV and other infectious diseases.

Whether there are differences in target engagement and downstream signaling of our CAR constructs remains to be investigated to further elucidate the mechanism of CARs binding linear *vs*. conformational epitopes. Overall, the fact that the 4D08-CAR showed similar killing kinetics on HBV-expressing target cells but lower background activation on control cells argues for moving forward with the 4D08-CAR in a clinical setting. A first proof-of-concept *in vivo* experiment showed that all CAR constructs have on-target activity, reduce the number of HBV-positive hepatocytes and cause a transient ALT increase due to hepatocyte killing. Injection of CAR-T cells was safe *in vivo* and could lower viral parameters in most mice chronically infected with AAV-HBV. However, a more thorough comparison of all three constructs *in vivo* at different CAR-T cell doses is warranted to further clarify the differences between the constructs. In the immunodeficient mice that were used to avoid a B- or T-cell response against the fully human CAR constructs (11), a much slower clearance of circulating viral antigens than in immunocompetent mice was expected. Thus, differences seen during the observation period were lower than those expected in immune competent individuals able to raise an antibody response that contributes significantly to clearing circulating antigen. In addition, starting values of HBeAg in the mock group were slightly higher than in CAR-treated groups. A longer experimental time frame and a higher dose of CAR-T cells would be beneficial to improve the antiviral effect.

In summary, we identified two high-affinity monoclonal human antibodies from single memory B cells of a donor with resolved HBV infection with broad neutralization capacity and reactivity against various HBV genotypes. These formed the basis for novel 2^nd^ generation chimeric antigen receptors rendering polyfunctional primary human T cells regarding cytokine secretion and cytotoxicity. This fast and efficient process of CAR generation from small quantities of cells from donors who efficiently controlled an infection can not only be used for hepatitis B but also for other infectious diseases. It will help broaden the availability of chimeric antigen receptors for adoptive T-cell therapy.

## Data availability statement

The original contributions presented in the study are included in the article/**Supplementary Material**. Further inquiries can be directed to the corresponding author.

## Ethics statement

Animal experiments were conducted in strict accordance with the regulations of the German Society for Laboratory Animal Science (GV-SOLAS) and the health laws of the Federation of European Laboratory Animal Science Associations (FELASA). Experiments were approved by the District Government of Upper Bavaria, permission number ROB-55.2-2532, Vet_02-23-104. Following 3R principles, the *in vivo* experiment was performed once. Mice were kept in a specific pathogen-free facility under appropriate biosafety levels, following institutional guidelines. The local ethics board of the University Hospital rechts der Isar of the Technical University of Munich approved using volunteer PBMC. All studies were conducted in accordance with the local legislation and institutional requirements. Written informed consent was obtained from all donors.

## Author contributions

SoS: Conceptualization, Data curation, Formal analysis, Investigation, Methodology, Project administration, Visualization, Writing – original draft, Writing – review & editing. LD: Conceptualization, Data curation, Formal analysis, Investigation, Methodology, Project administration, Visualization, Writing – original draft, Writing – review & editing. EL-V: Investigation, Writing – review & editing. TA: Investigation, Writing – review & editing. SF: Investigation, Writing – review & editing. WW: Investigation, Visualization, Writing – review & editing. TG: Investigation, Writing – review & editing. AC: Investigation, Visualization, Writing – review & editing. FeK: Investigation, Visualization, Writing – review & editing. CK: Supervision, Writing – review & editing. AK: Supervision, Writing – review & editing. SyS: Supervision, Writing – review & editing. SU: Conceptualization, Resources, Supervision, Writing – review & editing. FlK: Conceptualization, Resources, Supervision, Writing – review & editing. SR: Conceptualization, Resources, Supervision, Writing – review & editing. UP: Conceptualization, Data curation, Formal analysis, Funding acquisition, Investigation, Methodology, Project administration, Resources, Supervision, Writing – original draft, Writing – review & editing.
