# Supplementary material for: CARs derived from broadly neutralizing, human monoclonal antibodies identified by single B cell sorting target hepatitis B virus-positive cells
Source: Front Immunol. 2024 Apr 22;15:1340619. doi: 10.3389/fimmu.2024.1340619 (PMC11072186; doi:10.3389/fimmu.2024.1340619)
Supplement: Supplementary file 1 [file DataSheet_1.docx]

Supplementary Material

# Supplementary Figures


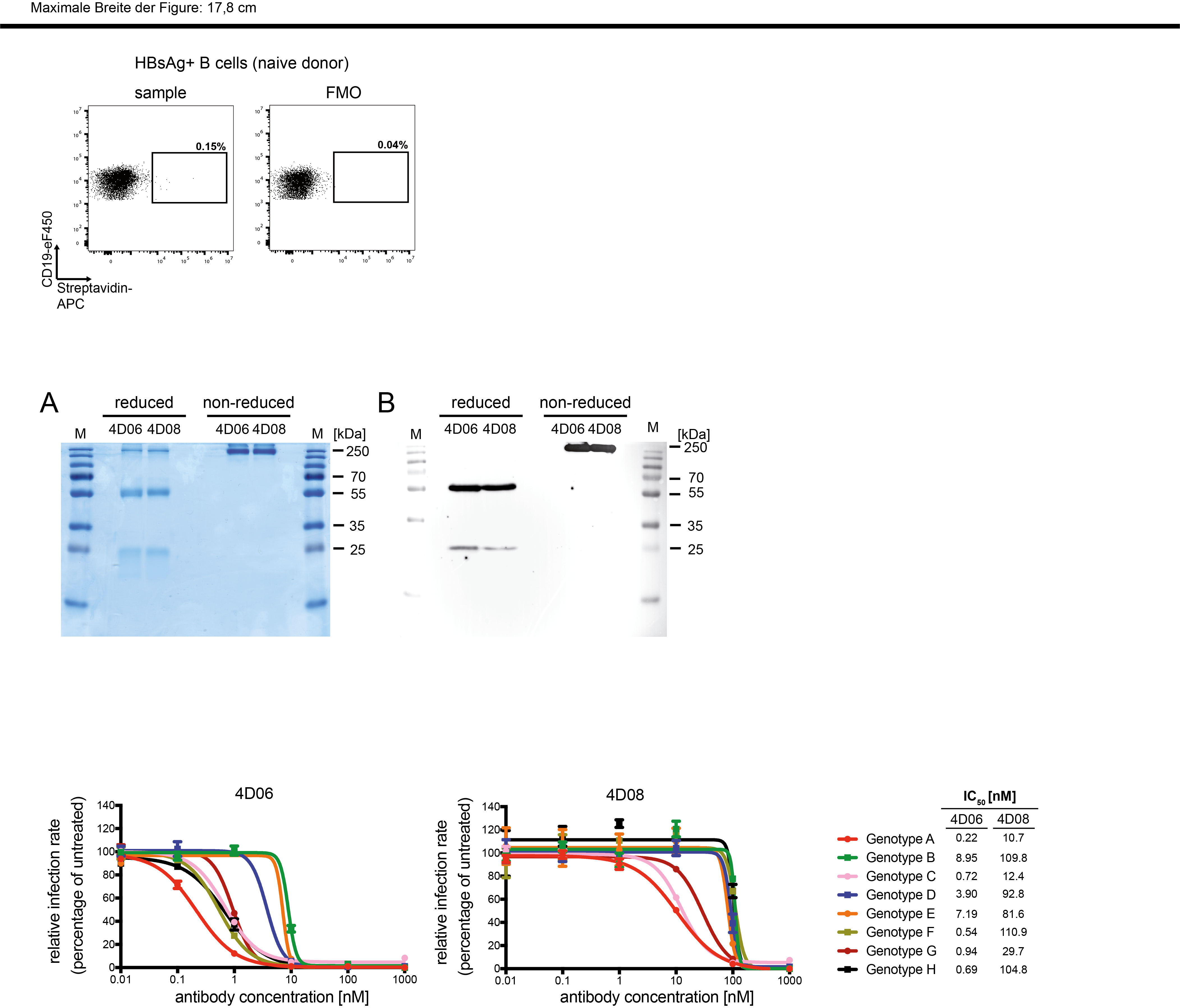


**Supplementary Figure 1: Staining of live CD19^+^ IgG^+^ B cells with HBsAg on B cells from naïve donor.** Frequencies refer to IgG^+^ cells.


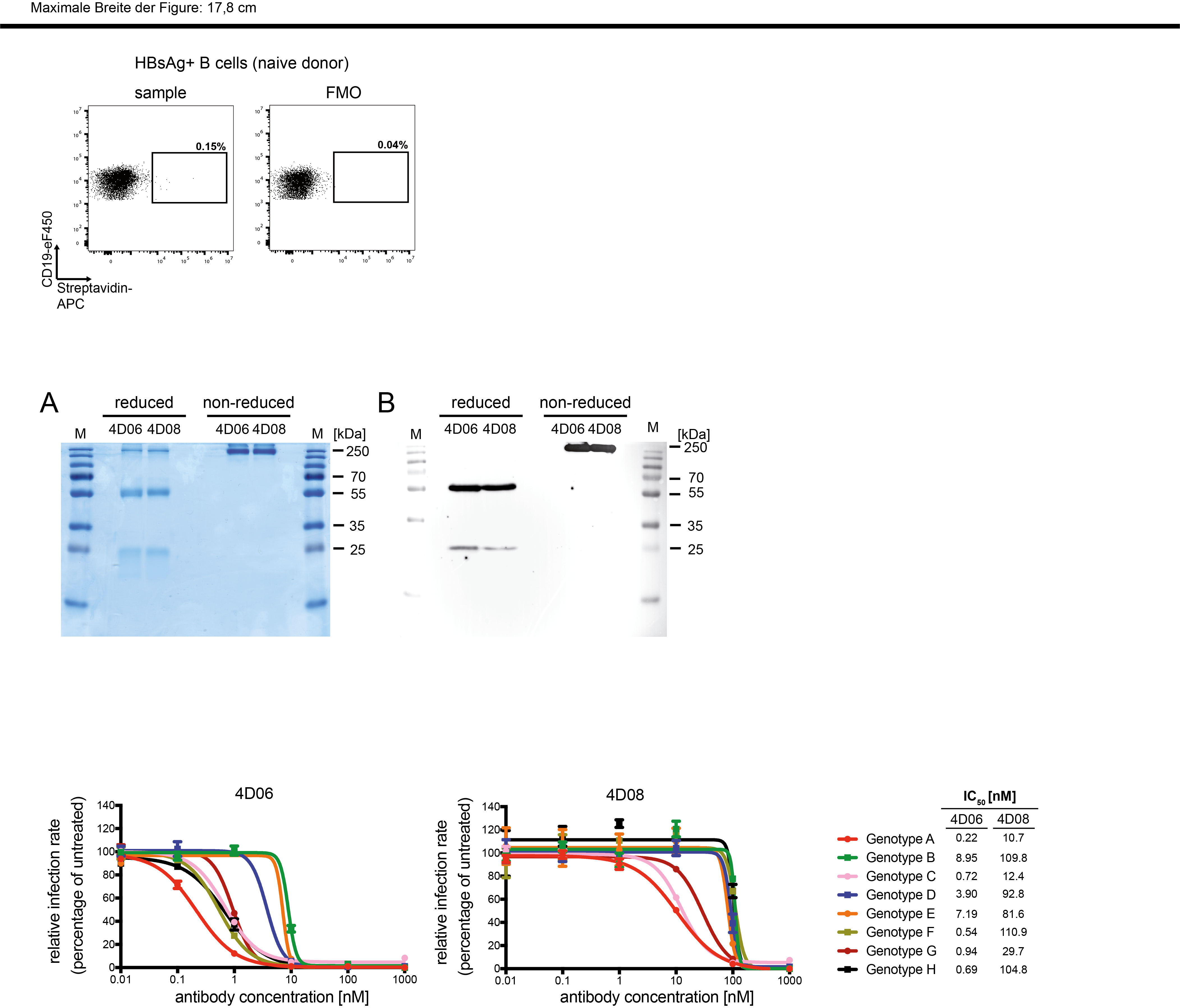


**Supplementary Figure 2: Assessment of purity of mAbs 4D06 and 4D08 after Protein G column affinity purification.** (A) 5 µg of the mAbs 4D06 or 4D08 was separated with PAGE under reducing and non-reducing conditions and total protein was stained with Coomassie Blue. (B) 0.1 µg of the indicated antibody was separated by PAGE under reducing and non-reducing conditions and wet-blotted to a PVDF membrane. After membrane blocking, antibodies were detected with polyclonal goat anti-human IgG HRP antibodies. Purity was > 95% for both constructs.

**
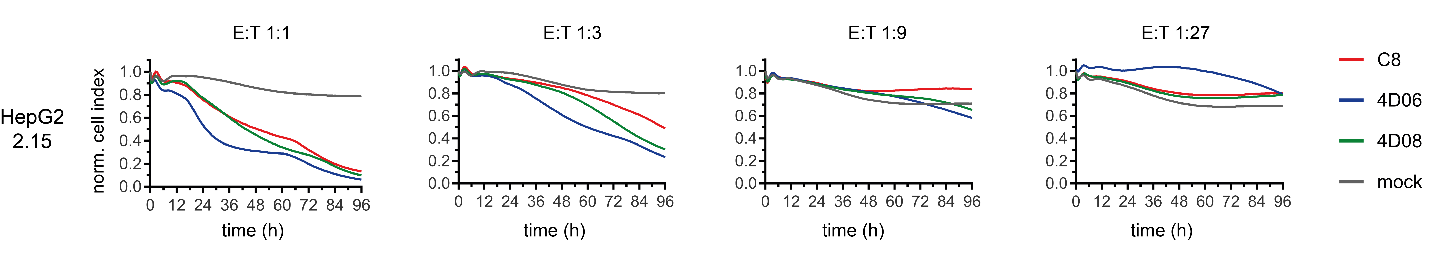
**

**Supplementary Figure 3:** xCELLigence cytotoxicity assay of the different CAR-transduced T cells co-cultured at various E:T ratios for 96h with HepG2-2.15 cells. Data points represent mean values from triplicate analyses.


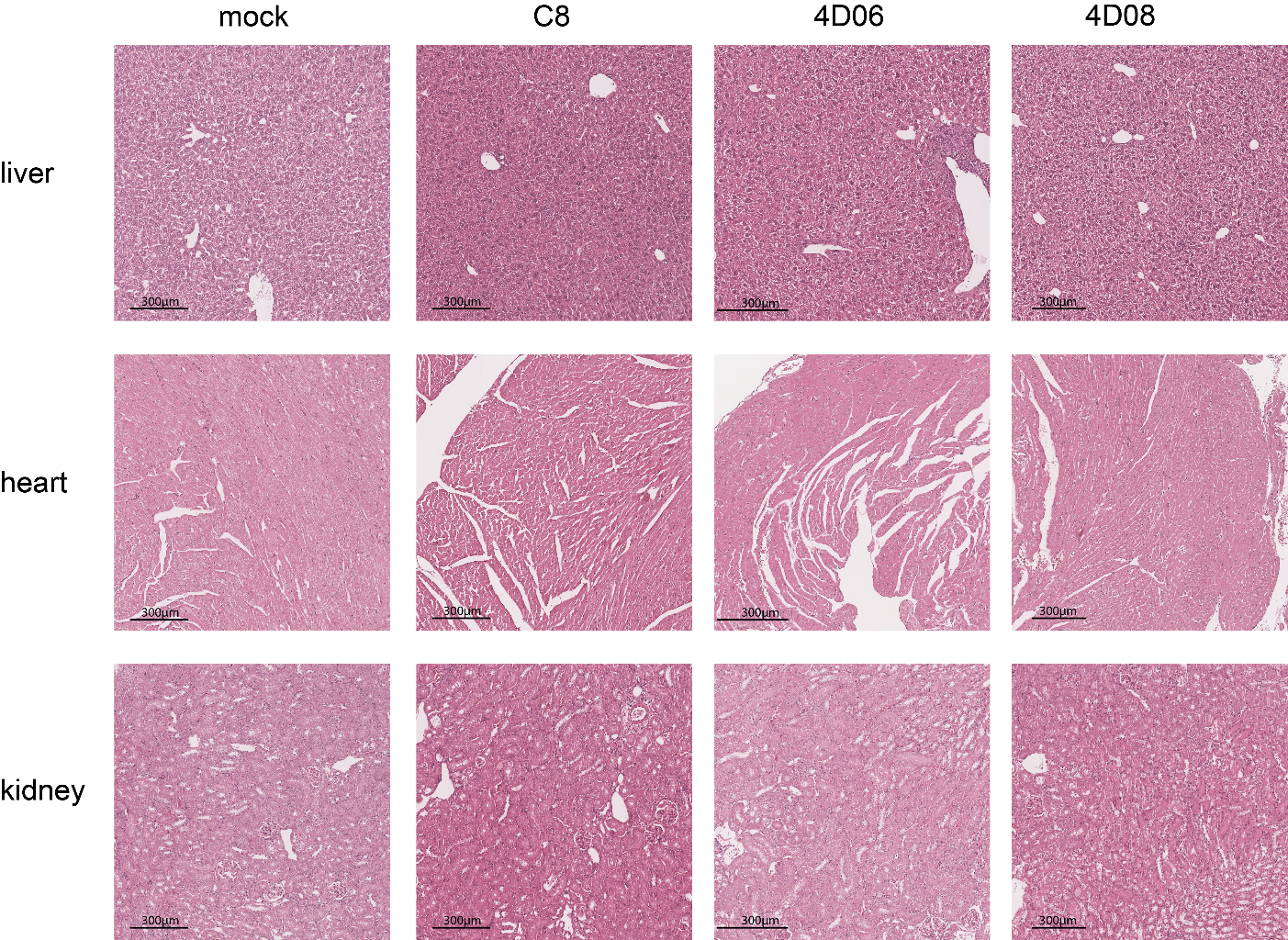


**Supplementary Figure 4:** Representative haematoxylin eosin stainings of liver, heart and kidney. Scale bar represents 300 μm.

**
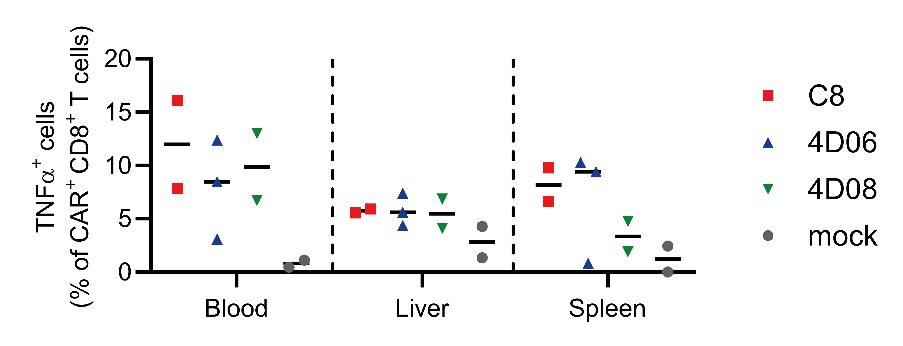
**

**Supplementary Figure 5:** Frequencies of TNFα-secreting CAR^+^ CD8^+^ T cells from blood, liver or spleen detected by intracellular cytokine staining after 16h *ex vivo* stimulation with plate-bound HBsAg. Symbols and lines represent individual mice.

# Supplementary Material & Methods

## Antibody production and purification

HEK293 cells were transiently transfected as described above in T150 tissue culture flasks. Cell culture supernatant was collected at day 3, 5 and 7 post-transfection and filtered with a 0.45 µm sterile filter. Purification was performed with a protein G column (GE Healthcare, Uppsala, Sweden) and low pH elution. Elution fractions were pooled, concentrated via filtration and submitted to a buffer exchange to PBS. Antibody concentrations after purification were determined via Bradford Assay with a bovine gamma globulin protein standard (Pierce, Thermo Fisher).

## SDS-PAGE and Western blot

Antibody purity was assessed with a 12% polyacrylamide gel electrophoresis (PAGE) under reducing and non‑reducing conditions followed either by Coomassie brilliant blue G250 (Carl Roth GmbH) staining or western blotting using a HRP-coupled goat anti-human IgG antibody (Sigma Aldrich) for detection. For Coomassie staining, 5 µg of each antibody was loaded to the gel, whereas for Western blot 100 ng were used. Under reducing conditions, the antibodies were heated for 10’ at 95°C in Laemmli sample buffer. Under non-reducing conditions antibodies were loaded to the gel diluted in non-reducing LDS sample buffer (Thermo Fisher).
